# Remote vibrotactile noise improves light touch sensation in stroke survivors’ fingertips via stochastic resonance

**DOI:** 10.1186/1743-0003-10-105

**Published:** 2013-10-11

**Authors:** Leah R Enders, Pilwon Hur, Michelle J Johnson, Na Jin Seo

**Affiliations:** 1 Department of Industrial and Manufacturing Engineering, University of Wisconsin-Milwaukee, Milwaukee, USA; 2 Department of Physical Medicine and Rehabilitation, Medical College of Wisconsin, Milwaukee, WI, USA; 3 Department of Occupational Science and Technology, University of Wisconsin-Milwaukee, Milwaukee, WI, USA

**Keywords:** Stroke, Sensory deficit, Biomechanics, Stochastic resonance, Noise

## Abstract

**Background and purpose:**

Stroke rehabilitation does not often integrate both sensory and motor recovery. While subthreshold noise was shown to enhance sensory signal detection at the site of noise application, having a noise-generating device at the fingertip to enhance fingertip sensation and potentially enhance dexterity for stroke survivors is impractical, since the device would interfere with object manipulation. This study determined if remote application of subthreshold vibrotactile noise (away from the fingertips) improves fingertip tactile sensation with potential to enhance dexterity for stroke survivors.

**Methods:**

Index finger and thumb pad sensation was measured for ten stroke survivors with fingertip sensory deficit using the Semmes-Weinstein Monofilament and Two-Point Discrimination Tests. Sensation scores were measured with noise applied at one of three intensities (40%, 60%, 80% of the sensory threshold) to one of four locations of the paretic upper extremity (dorsal hand proximal to the index finger knuckle, dorsal hand proximal to the thumb knuckle, dorsal wrist, volar wrist) in a random order, as well as without noise at beginning (Pre) and end (Post) of the testing session.

**Results:**

Vibrotactile noise of all intensities and locations instantaneously and significantly improved Monofilament scores of the index fingertip and thumb tip (*p* < .01). No significant effect of the noise was seen for the Two-Point Discrimination Test scores.

**Conclusions:**

Remote application of subthreshold (imperceptible) vibrotactile noise at the wrist and dorsal hand instantaneously improved stroke survivors’ light touch sensation, independent of noise location and intensity. Vibrotactile noise at the wrist and dorsal hand may have enhanced the fingertips’ light touch sensation via stochastic resonance and interneuronal connections. While long-term benefits of noise in stroke patients warrants further investigation, this result demonstrates potential that a wearable device applying vibrotactile noise at the wrist could enhance sensation and grip ability without interfering with object manipulation in everyday tasks.

## Introduction

Many of 7 million stroke survivors in the U.S. [1] experience not only motor deficit [2-4] but also sensory deficits [5] especially in the hand. Carey and Matyas [6] found that discriminatory sensory loss was observed in almost 50% (24 of 51 subjects) in chronic stroke survivors, compared to almost 85% (57 of 67 subjects) of acute stroke survivors [7]. Turton and Butler [8] found in a case study that a stroke survivor had a decreased ability to correctly identify the time and locations of stimuli applied to both the palm and digits of the affected hand [8]. When the stroke subject was asked to correctly identify where and when a touch stimulus was applied on their hand, the subject only responded to the tests correctly about 65% of the time [8].

While tactile sensation is critical for hand function, current stroke rehabilitation practices predominantly focus on motor re-training with limited emphasis on sensory re-training and sensorimotor integration. Cutaneous sensory feedback is essential for dexterity, fine finger movements, grip stability, and the setting and maintenance of force production during grip and object manipulation [9,10]. For instance, tactile sensory feedback from receptors in the fingertips is used for motor adaptation to surface characteristics [11] and dexterous hand movement [12]. Tactile sensory deficit experienced by stroke survivors can lead to inappropriate grip force regulation and inefficient safety margins [13]. The reduced sensory feedback experienced in stroke survivors may deteriorate feedback control of finger forces leading to unstable grip and object slipping against the finger, thereby hampering their hand grip function. Therefore, it is necessary to improve tactile sensation for stroke survivors, which may facilitate rehabilitation to improve dexterity, finger force control, and thus, hand function.

Previous research has aimed at increasing tactile sensation through a range of modalities. Anesthetic cream to the forearm has been shown to increase fingertip tactile sensation for healthy individuals [14] and stroke survivors [15] by inducing short-term changes in cortical representations [15]. Intense sensory retraining for chronic stroke survivors through repetitive sensory exercises (i.e. shape and texture discrimination) over a period of several weeks has also shown some potential to increase tactile sensation [16-18].

Stochastic resonance is a phenomenon in which addition of noise (e.g., vibrotactile noise) to a weak signal maximizes the detection and transmission of the weak signal [19-21]. Collins et al. [22] found that healthy individuals’ tactile sensation can be improved with certain levels of subthreshold vibrotactile noise (below the level at which a person can perceive the vibration), while it can be degraded if noise is too high (i.e., suprathreshold) “masking” the original signal. Therefore, intensity of noise should be high enough for the signal to cross the threshold but low enough not to swamp the signal and decrease the signal to noise ratio [20,22,23]. Previous work has shown optimum vibrotactile noise intensity as low as 50% of the sensory threshold for sensing a vibration at the fingertips [23,24], while others have shown as high as 90% of the sensory threshold to be effective [19,21,25]. No consensus has been reached regarding the optimum vibrotactile noise intensity, especially for stroke survivors.

In light of the accumulating evidence for stochastic resonance, a wearable device applying vibrotactile noise to the fingertip has been developed by Kurita et al. [24]. While the device improves tactile sensation at the fingertip pad, a noise-generating device placed at the lateral aspect of the fingertip adversely interferes with object manipulation and dexterous finger movement by blocking physical contact between the finger and object, thus defeating the purpose of somatosensory enhancement. Furthermore, donning and doffing an assistive glove is difficult for stroke survivors, especially those with spasticity [26,27]. Thus, the desirable design would involve remote application of the vibrotactile noise to a location on the back of the hand or wrist that can still enhance tactile sensation. However, it is unknown if remote vibrotactile noise (i.e., away from the fingertip) could influence tactile sensation of the fingertip. In this study, we investigated how vibrotactile noise applied to various noise locations proximal to the fingertips could influence tactile sensation of the fingertip for stroke survivors.

The main objective of this study was to determine the effect of remote subthreshold vibrotactile noise on the tactile sensation of the index and thumb fingertips in stroke survivors. To achieve this objective, subthreshold vibrotactile noise was applied to one of four locations on the paretic upper limb (dorsal hand proximal to the index finger knuckle, dorsal hand proximal to the thumb knuckle, dorsal wrist, or volar wrist) at one of three noise intensities (40%, 60%, or 80% of the sensory threshold). It was hypothesized that remote subthreshold vibrotactile noise improves light touch sensation and spatial discrimination at the index and thumb fingertip pads in stroke survivors.

## Methods

### Subjects

Ten chronic stroke survivors (mean age ± SD = 60 ± 9 years) with sensory deficit participated in this study (Table 1). Hand motor function, evaluated using the hand and wrist subdivision of the Fugl-Meyer Assessment [28] (Table 1), was 19 ± 5 (out of a possible 24). All stroke survivors were at least 6 months post stroke. Subjects with history of upper extremity orthopedic conditions were excluded from this study. Subjects’ tactile sensory deficit was recorded with the Semmes-Weinstein Monofilaments [29] and the Two-Point Discrimination Tests [29] for the index finger and thumb. Sensory deficit was defined as abnormal scoring for either of the sensory tests for either the index finger or the thumb. All subjects signed a consent form and followed a protocol approved by the Institutional Review Board.

**Table 1:** Subject demographic information

**Subject**	**Gender**	**Age**	**Fugl**-**Meyer**	**Monofilament (mm)**		**Two Point Discrimination (mm)**		**Vibrotactile sensory threshold (A peak to peak)**			
				**Index**	**Thumb**	**Index**	**Thumb**	**Dorsal hand proximal to index knuckle**	**Dorsal hand proximal to thumb knuckle**	**Volar wrist**	**Dorsal wrist**
1	F	67	9	3.61	3.61	5	6	0.19	0.07	0.17	0.20
2	F	75	24	3.61	3.61	5	6	0.75	0.05	0.14	0.17
3	M	71	13	3.61	3.61	6	8	0.20	0.12	0.20	0.20
4	M	57	23	3.61	3.61	4	3	0.21	0.13	0.19	0.19
5	M	52	21	3.61	3.61	4	5	0.20	0.11	0.15	0.06
6	F	60	16	6.65	6.65	15	10	0.09	0.07	0.19	0.21
7	F	60	22	3.61	3.61	5	5	0.08	0.05	0.14	0.19
8	F	47	20	3.61	3.61	3	5	0.17	0.06	0.07	0.15
9	M	54	24	3.61	3.61	4	4	0.09	0.07	0.07	0.17
10	M	59	16	3.61	3.61	4	5	0.20	0.12	0.16	0.20

Demographic information of all subjects with the baseline sensory and motor function scores and vibrotactile sensory thresholds.

### Procedure

Subjects’ Monofilament and Two-Point Discrimination scores for the index and thumb fingertips were compared with and without noise. Specifically, sensory scores without noise were recorded at the beginning (pre) and end (post) of the testing session. Sensory scores for the pre and post test were compared to ensure no learning effect and no residual effect of noise after the exposure during the one day testing session. In between the pre and post sensory tests without noise, sensory scores with noise were recorded while subthreshold vibrotactile noise was applied to four different locations at three noise intensities. The subthreshold vibrotactile noise was turned on immediately before each sensory test and was turned off immediately after each sensory test (lasting approximately 1 minute each). The testing session lasted for approximately two hours for each subject.

**Figure 1:**
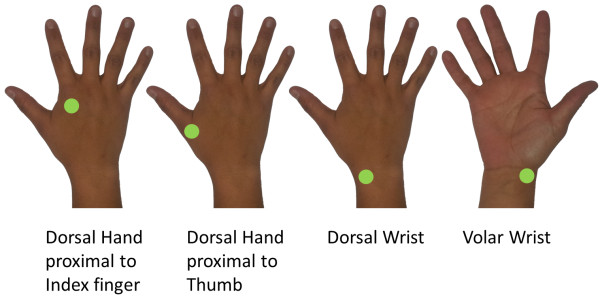
**Vibrotactile noise locations.** Sensation scores were recorded while remote vibrotactile noise was applied to one of four locations: 1) dorsal hand approximately 2 cm proximal to the index finger knuckle; 2) dorsal hand approximately 2 cm proximal to the thumb knuckle; 3) dorsal wrist, medial to the radial styloid process; and 4) volar wrist, medial to the radial styloid process. Noise intensity was set to 40%, 60%, or 80% of the sensory threshold for each location for each stroke survivor.

Subthreshold vibrotactile noise was white noise bandwidth filtered at 0 to 500 Hz, applied with a C-3 Tactor (Engineering Acoustics, Inc. Casselberry, Florida). Due to the characteristics of the C-3 Tactor, the vibration amplitude could have been larger for 100-300 Hz which includes the sensitive range of the Pacinian corpuscles. The noise was applied to one of four locations in the paretic upper limb (Figure 1): 1) dorsal hand approximately 2 cm proximal to the index finger knuckle; 2) dorsal hand approximately 2 cm proximal to the thumb knuckle; 3) dorsal wrist, medial to the radial styloid process; and 4) volar wrist, medial to the radial styloid process. These locations were arbitrarily chosen with the intention of developing a future wearable rehabilitation device for stroke survivors. Since the long-term goal of the research is to improve dexterity and grip control, noise locations that would interfere with gripping, such as the fingertip or palm, were avoided. Presentation of noise locations was block randomized.

Noise intensities were set to 40%, 60%, or 80% of the sensory thresholds specific for each location. The order of testing different noise intensities was randomized within each location block. To determine the sensory threshold, the noise intensity was increased and decreased until the subject was barely able to distinguish between an “off” and an “on” presentation of the vibrotactile noise (i.e., the method of ascending and descending limits [22]). Subjects’ mean sensory threshold occurred when the Tactor was driven by current of 0.17 A peak-to-peak (Table 1). There is a linear relationship between the current and amplitude of the vibration. According to the data sheet from the manufacturer, 0.17 A peak-to-peak corresponds to a maximum amplitude of 260 μm. Subthreshold noise intensities were chosen not only so that subjects could not distinguish between trials with and without noise [21], but also because suprathreshold noise has been shown to degrade performance [23].

The Monofilament and Two-Point Discrimination Tests were administered using standard testing measures. For the Monofilament score, beginning with the baseline 2.83 Monofilament (indicating the threshold for “normal sensing”), the Monofilament was applied to the fingertip at least three times and the smallest Monofilament for which the subjects responded “yes” and could identify the correct finger that was touched marked the score [29]. The Two-Point Discrimination test was conducted so that subjects were asked to respond either “one” for a single point and “two” for two points separated by a small distance [29]. One and two point stimuli were alternated randomly. The smallest distance where the subjects responded correctly three consecutive times to identifying two separated points was used for their Two-Point Discrimination score. A score of 2.83 [30] and 5 mm [31] was considered normal for the Monofilament test and Two-Point Discrimination tests, respectively.

### Data analysis

Monofilament Test scores (ranging from 2.83 to 6.65) were converted to the corresponding estimated logarithmic bending force (ranging from .07 to 300 grams) for the statistical analysis. Paired T-Tests showed that neither the Monofilament score nor Two-Point Discrimination Test score without vibrotactile noise at the beginning of the testing session was significantly different from that at the end of the testing session without noise (*p* = .33 and *p* = .78 for the Monofilament and Two-Point Discrimination, respectively), indicating that there was no learning effect with repeated sensory tests and there was no residual effect of noise on tactile sensation. Therefore, sensory scores pre and post testing sessions were averaged to become the noise off trials.

Two separate repeated measures ANOVAs using Minitab statistical software (Minitab Inc., State College, Pennsylvania, USA) were completed to determine how stroke survivors’ tactile sensation varied with vibrotactile noise. The first ANOVA determined if stroke subjects’ Monofilament Test scores varied significantly by noise ‘on’ and ‘off’ , noise location (nested in the noise ‘on’ condition), noise intensities (nested in noise ‘on’), finger (index or thumb), and their second-order interactions. The same ANOVA was performed for the Two-Point Discrimination Test scores. Specifically, these two ANOVAs were used to determine 1) if noise had an overall effect on the Monofilament and Two-Point Discrimination Test scores, and 2) if different noise locations and intensities had varying effects on the Monofilament and Two-Point Discrimination Test scores. Since the Test for Skewness showed skewed Monofilament (*p* < .01) and Two-Point Discrimination score data (*p* < .01) [32], log and inverse (1/x) transformations were applied to the Monofilament and Two-Point Discrimination data, respectively, to yield non-significant skew values. Transformed data were used for the ANOVAs. As an additional analysis, a Pearson Correlation examined the relationship between improvement in sensation and functional motor score (Fugl-Meyer Assessment).

## Results

### Improved monofilament scores with remote subthreshold vibrotactile noise

**Figure 2:**
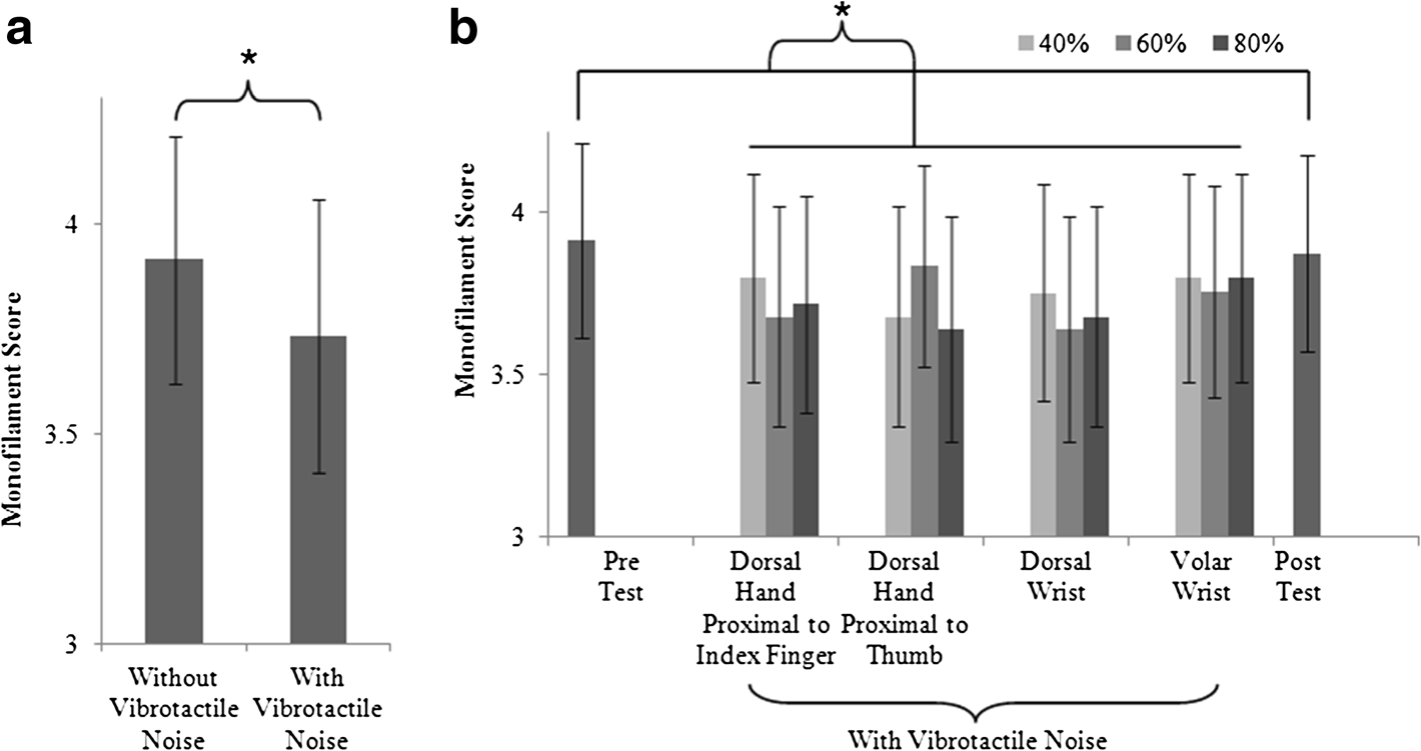
**Monofilament Scores with and without subthreshold vibrotactile noise.** Mean ± SE Monofilament scores significantly decreased with subthreshold vibrotactile noise (noise locations, intensities, fingers, and subjects pooled) (*p* < .01) **(a)**. Noise locations and intensities did not significantly affect the improvement of Monofilament score (fingers and subjects pooled, *p* > .05 for noise location and intensity) **(b)**.

Stroke survivors’ fingertip mean Monofilament Test scores improved from 3.91 to 3.73 when vibrotactile noise was applied to the paretic hand remotely from the fingertip (subject, noise location, intensity, and fingers pooled) (Figure 2, Additional file 1). Seven out of the ten stroke survivors had improved Monofilament Test score when vibrotactile noise was applied to the paretic hand remotely from the fingertip, for at least one noise location, noise intensity, and finger. The improvement in the Monofilament scores with vibrotactile noise was statistically significant (ANOVA, F_1,245_ = 14.3, *p* < .01). All other effects of noise location (*p* = .13), intensity (*p* = .48), finger (*p* = .45), and interactions were not significant (*p* > .05). Monofilament scores improved from mean ± standard deviation of 3.91 ± 0.94 to 3.73 ± 1.03 with vibrotactile noise (subject, noise location, intensity, and fingers pooled). Neither finger (index, thumb) nor the interaction between finger and noise was significant, indicating vibrotactile noise improved light touch sensation for both fingers. Noise location and intensities did not significantly affect the Monofilament scores, indicating that all remote vibrotactile noise at all intensities improved Monofilament score at the fingertips to the similar degree. As described earlier, monofilament scores without vibrotactile noise did not change pre vs. post test (*p* = .33), indicating no learning effect and no after-effect of noise. Improvement in the Monofilament score with noise was not significantly related to the Fugl-Meyer score (Pearson Correlation, p = .84).

### No significant effect of vibrotactile noise on Two-Point Discrimination

**Figure 3:**
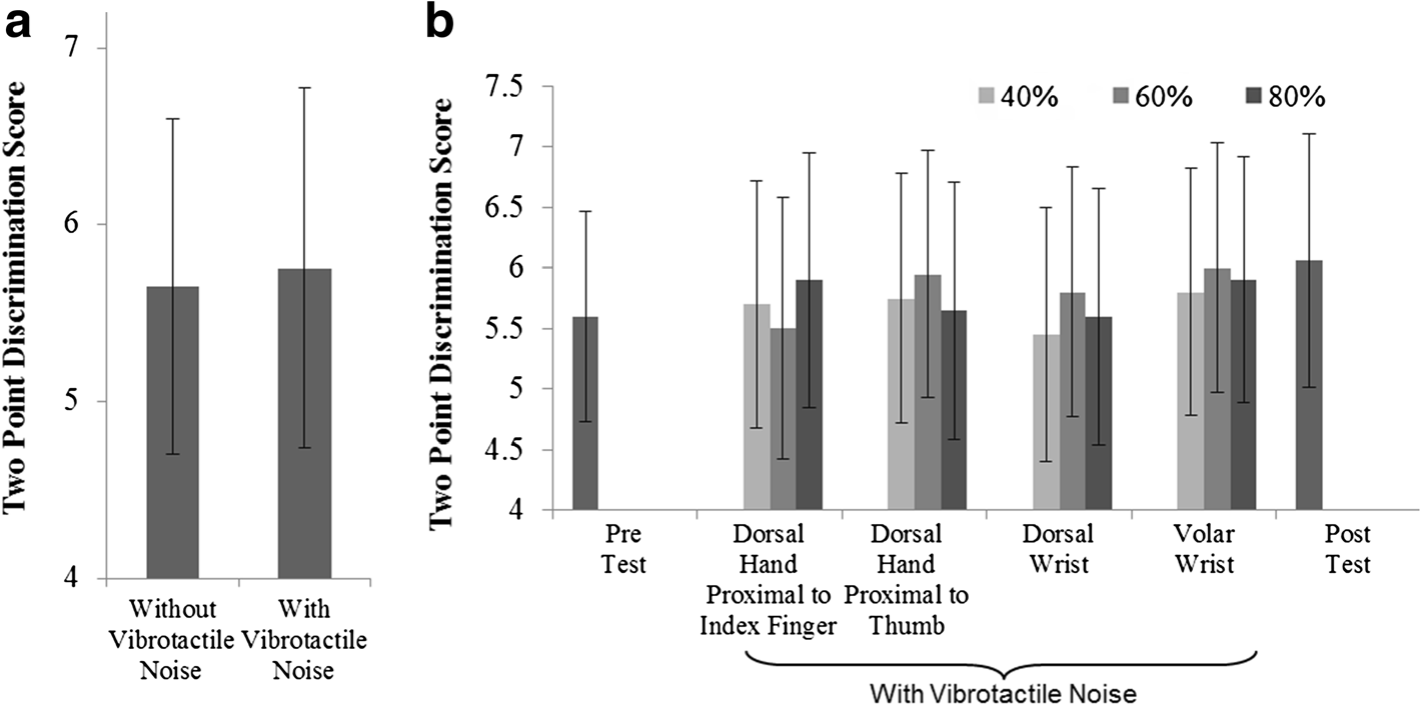
**Two**-**Point Discrimination scores with and without subthreshold vibrotactile noise.** Mean ± SE Two Point Discrimination scores were not significantly affected by the vibrotactile noise **(a)** nor with noise locations, intensities, fingers, and their interactions (fingers and subjects pooled) (*p* > .05) **(b)**. The Two-Point Discrimination score without vibrotactile noise did not change at the beginning vs. end of the testing session.

Stroke survivors’ Two-Point Discrimination Test score did not significantly change when vibrotactile noise was applied to the paretic wrist and dorsal hand (ANOVA, F_1,245_ = .04, p = .84, Figure 3, Additional file 2). Mean Two-Point Discrimination was significantly dependent upon finger (*p* < .01) and was significantly higher for the thumb compared to the index finger. Mean Two-Point Discrimination scores were not significantly dependent upon noise intensity (*p* = .82), location (*p* = .19), or any interactions (*p* > .05). The Two-Point Discrimination scores without vibrotactile noise did not change pre vs. post test (*p* = .78).

## Discussion

### Remote subthreshold vibrotactile noise enhanced stroke survivors’ light touch sensation at the fingertips

Light touch sensitivity at the pads of the thumb and index fingertips was enhanced with the subthreshold vibrotactile noise at the wrist or dorsal hand, as evidenced by the improved Monofilament Test score (Figure 2). All noise intensities (40%, 60%, and 80% of the sensory threshold) and locations (dorsal hand and wrist) improved the fingertip light touch sensation. The benefit of the subthreshold vibrotactile noise was instantaneous, and not influenced by learning or after-effect of noise (as evidenced by insignificant difference between the Monofilament scores without noise pre and post test). The largest improvement of 25% in Monofilament score with vibrotactile noise compared to without vibrotactile noise was found for the vibrotactile noise at the dorsal wrist at 60% of the sensory threshold and for the vibrotactile noise at the dorsal hand proximal to the thumb knuckle at 80% of the sensory threshold (Figure 2b). Hand motor function (as measured by the hand and wrist subdivision of the Fugl-Meyer assessment) was not found to be related to the degree of sensory improvement. Therefore, stochastic resonance improved sensation for the stroke survivors in this study who ranged from 9 to 24 (out of 24) in hand motor function levels.

The clinical implication of this finding is significant. This study finding indicates that a wearable assistive wrist band applying subthreshold vibrotactile noise can be developed to enhance touch sensation for stroke survivors’ fingertips and assist with their dexterous hand movement. The advantage of this wearable assistive wrist band compared to the current glove with a vibrator attached at the fingertip [24] is that the wrist band minimally interferes with manual dexterity of stroke survivors. In addition, the vibration is minute at the level that is not perceivable. Thus, this vibration is unlikely to result in numbness or tissue damage in the long-run.

### Potential mechanisms of remote sensory enhancement

It is unlikely that the light touch sensation improved via the vibrotactile noise traveling from the wrist or dorsal hand to the fingertips through the skin, because vibration significantly attenuates across the skin. In general, vibration can improve tactile sensation by directly stimulating the tactile receptors in the finger skin [24]. However, Kurita et al. [24] reported that mechanical vibration may lose 90% of its original power when it travels 1 to 2 cm on the skin [24]. In our study, the distance between the fingertip and noise locations ranged from 10 to 20 cm. Therefore, it is unlikely that the index and thumb fingertips’ sensation would have been affected by transfer of the mechanical vibration through the skin from any of the noise locations to the thumb or index fingertip.

A more likely mechanism for enhanced light touch sensation at the fingertips with remote vibrotactile noise is that the vibrotactile noise at the wrist and dorsal hand may have increased the sensory neurons’ excitability not only for the wrist and dorsal hand but also for the fingertips through interneuronal connections either in the spinal or supraspinal level. For example, Merzenich et al. [33] found that median, ulnar, and radial nerves, although peripherally separate, appear to be directly connected in the central nervous system. Specifically, they have shown that immediately after median nerve transaction, significant inputs from the dorsum of the hand (innervated by the radial and ulnar nerve) appear in the somatosensory cortex area that was previously innervated by the median nerve in monkeys. Such emergence of radial and ulnar nerve representation in the median nerve territory in the somatosensory cortex was immediate, suggesting pre-existing synaptic connections between the sensory representations of the palmar and dorsal areas of the hand [33]. Unmasking of the pre-existing connections has been shown in other studies involving healthy persons [14] as well as people with stroke [15]. In addition to the interneuronal connections in the central nervous system between the palmar and dorsal areas of the hand, it has been shown that vibrotactile noise results in increased cortical as well as spinal neuronal activities in humans and cats, which demonstrates the effect of stochastic resonance in the central nervous system [34,35]. Therefore, vibrotactile noise applied to the wrist or dorsal hand may have increased the fingertip sensation by increasing the excitability of the sensory neurons in the central nervous system through stochastic resonance and interneuronal connections.

Another potential mechanism for the enhanced light touch sensation is that vibrotactile noise at the wrist or the dorsal hand may have increased the synchronization of sensory neuron firing between the spinal cord and the somatosensory cortex [34,35]. The increased synchronization may facilitate neural communication between the spinal and cortical levels [36], thereby enhancing detection of light touch stimulation from the fingertips to the somatosensory cortex.

### Lack of noise effect on Two-Point Discrimination

Two-Point Discrimination sensation was not significantly affected by the subthreshold vibrotactile noise in this study (Figure 3). This finding aligns with a study done by Kurita et al. [24] that subthreshold vibrotactile noise enhanced only light touch sensation but not Two-Point Discrimination at the fingertips. A reason for inconsistent results may be that the Monofilament Test and Two-Point Discrimination Test assess different aspects of sensation. The Monofilament Test assesses the threshold of the mechanoreceptors responsible for pressure, whereas the Two-Point Discrimination Test examines spatial resolution of receptive fields for discriminative touch [37]. Therefore, the present study’s finding suggests that spatial resolution of mechanoreceptors was not affected by the subthreshold vibrotactile noise.

### Limitations and future work

One limitation of this study could be the use of the Two-Point Discrimination Test to demonstrate impact on the tactile spatial resolution. Although still used widely in clinics to demonstrate a deficit in spatial acuity, the Two-Point Discrimination has been criticized previously by scientists for the response variable of “one point” or “two points” as an unreliable outcome measure that has high variability both between and within subjects [38]. Additionally, although Monofilament Test Score showed that all subjects had light touch deficit at the beginning, not all subjects had sensory deficit according to the Two-Discrimination Test. Therefore, the lack of improvement in the Two-Point Discrimination Test with vibrotactile noise could have been due to near-normal starting scores leaving not much room for improvement.

Additionally, this study is limited by examining the effect of remote stochastic resonance on sensation from only two fingers, the index and thumb fingers. Due to limited time to examine each noise level and location, no additional fingers were examined for sensation. As discussed earlier, remote stochastic resonance (at sites on the hand/wrist innervated by the radial nerve) may have influenced both index and thumb fingertip sensation through integration of information from the median, ulnar, and radial nerves in the central nervous system. It can only be postulated that similar improvements found with the index and thumb fingertip may also occur for the middle, ring, and little fingertips through this integration. However, further testing would be necessary to verify.

In this study, the Monofilament scores were recorded from a set of 5 Monofilaments, instead of the set of 20 Monofilament sizes. The 20 Monofilament sizes would have shown greater resolution to the degree of sensory improvement. However, the 5 Monofilament set was still sufficient to show the large changes in sensation for this study.

While the present study demonstrated the immediate effects of vibrotactile noise on sensory enhancement, in order to be applied to a longer term sensorimotor rehabilitation therapy, future studies need to examine the effects of repeated exposure and the long-term benefits of vibrotactile noise in stroke survivors. Although Monofilament scores pre and post the 2-hour test were not significantly different in the present study, longer or repeated exposure to the vibrotactile noise may elicit longer-lasting improvements in fingertip sensation. A sensory re-training program, such as the one described by Carey et al. [17], could be complimented by the addition of vibrotactile noise. Furthermore, the effect of sensory enhancement on motor function following stroke should be investigated. Specifically, how effectively the enhanced sensation at the fingertips leads to improved dexterity such as precise grip force regulation and coordination [13,39] could be investigated. Finally, a prototype of a vibrotactile noise wrist band will be developed for clinical evaluation to determine the efficacy of the remote vibrotactile noise for rehabilitation post stroke.

## Conclusions

Remote stochastic resonance phenomenon was investigated to determine if subthreshold vibrotactile noise at the wrist or dorsal hand can enhance the tactile sensation at the fingertip of the stroke survivors. The application of the subthreshold vibrotactile noise at the wrist and dorsal hand instantaneously enhanced the light touch sensation at the fingertip of stroke survivors. This benefit in the light touch sensation was not influenced by learning effect. The most improvement in the light touch sensation at the fingertip occurred when the dorsal wrist and the dorsal hand proximal to the thumb knuckle were stimulated at 60% and 80% of the sensory thresholds, respectively. This study carries clinical significance, since the finding of this study demonstrates strong potential that a subthreshold vibrotactile noise-generating assistive wrist band may be able to enhance fingertip tactile sensation for stroke survivors and may contribute to enhanced manual dexterity and abilities for activities of daily living.

## Competing interests

Dr. Seo and Leah Enders are named inventors on a provisional patent application filed by the UWM Research Foundation on behalf of the University of Wisconsin – Milwaukee and related to a device based on remote subthreshold vibrotactile noise.

## Authors’ contributions

LE carried out the design of the study, data collection, and data analysis, drafted and revised the manuscript. PH participated in data analysis, drafted and revised the manuscript. MJ participated in data analysis and revised the manuscript. NS conceived and coordinated the study, analyzed the data, and revised the manuscript. All authors read and approved the final manuscript.

## Supplementary Material

Additional file 1 Monofilament Score Raw Data, Individual stroke survivors’ Monofilament scores with and without subthreshold vibrotactile noise. Click here for file

Additional file 2 Two-Point Discrimination Score Raw Data, Individual stroke subjects’ Two-Point Discrimination scores with and without subthreshold vibrotactile noise. Click here for file
